# Association of human leukocyte antigen-DRB haplotype in multiple sclerosis population of Khuzestan, Iran

**Published:** 2018-10-07

**Authors:** Nooshin Delfan, Hamid Galehdari, Mohammad Shafiei, Farideh Ghanbari-Mardasi, Tahereh Latifi, Nastaran Majdinasab, Tahereh Seifi

**Affiliations:** 1 Department of Genetics, School of Sciences, Shahid Chamran University of Ahvaz, Ahvaz, Iran; 2 Multiple Sclerosis Society, School of Rehabilitation, Ahvaz Jundishapur University of Medical Sciences, Ahvaz, Iran

**Keywords:** Multiple Sclerosis, Human Leukocyte Antigen-DRB5, Human Leukocyte Antigen-DRB1*1501, Polymerase Chain Reaction, Iran

## Abstract

**Background:** One of the demyelinating and inflammatory diseases of the central nervous system (CNS) is multiple sclerosis (MS). Though pathogenesis of MS is still unknown, both genetic and environmental factors are involved. The human leukocyte antigen (HLA) class-II alleles including HLA-DRB5*01, DQB1*0602, DRB1*1501, and DQA1*0102 may have remarkable effect in MS risk although it is controversial in studies. As there is no data with respect to the HLA-DRB1*1501-DRB5*01 correlation with MS in Khuzestan Province, Iran, the goal of the survey was to investigate the association of this haplotype with MS in this population.

**Methods:** The study focused on DRB5*01-DRB1*1501 haplotype association with MS in 200 patients and 200 healthy individuals. Typing of HLA was carried out by polymerase chain reaction (PCR) amplification with sequence-specific primers (SSP) method. SPSS software was used for the statistical analyses.

**Results: ** No association between DRB5*01^+^-DRB1*1501^+^ and MS was found (P = 0.156). Distribution of DRB1*1501^+^-DRB5*01^-^ (carrying DRB1*1501^+^ but not DRB5*01^-^) and DRB1*1501^-^-DRB5*01^-^ haplotypes was statistically different between patients and controls (29.73% vs. 11.81%, P < 0.001) and (42.16% vs. 68.50%, P < 0.001), respectively. However, DRB1*1501^-^-DRB5*01^+^ revealed no association with MS (15.13% vs. 11.81%, P = 0.403). HLA-DRB1*1501^-^-DRB5*01^+^ was signiﬁcantly more frequent among female patients with MS (16.19% vs. 6.12%, P = 0.019) and Persian group (17.11% vs. 5.79%, P = 0.027). Positive correlation of HLA-DRB1*1501^+^-DRB5*01^-^ haplotype with the expanded disability status scale (EDSS) steps from 5 to 10 was observed (62.50% vs. 25.76%, P = 0.026). Moreover, no meaningful association was shown among the haplotypes with EDSS, course of MS, ethnicity, and gender.

**Conclusion:** Our findings suggest that DRB1*1501^+^-DRB5*01^-^ and DRB1*1501^-^-DRB5*01^-^ haplotypes may have positive association with MS risk. Also, this survey indicates that HLA-DRB1*1501^-^-DRB5*01^+^ is involved in susceptibility of the disease among women and Persians. DRB1*1501^+^-DRB5*01^-^ genotype frequency may have a key role in MS developing.

## Introduction

Multiple sclerosis (MS) manifests with axonal degeneration and inflammation. MS is a chronic demyelinating disease of the central nervous system (CNS). Numbness, bladder dysfunction, paresis, visual disturbance, and others are known as the major characteristics of the disease. However, the precise cause of it is still unclear. It is known as a multifactorial disease because both genetic and environmental factors have been shown to contribute to the pathogenesis. Twin studies have indicated that genetic factors play an important role in MS susceptibility.^1^^,^^2^

Almost 45 years ago, MS association with the major histocompatibility complex (MHC) on chromosome 6p21.3 was identiﬁed. In particular, MS association with the human leukocyte antigen (HLA) genes from the MHC class II, DRB1*1501 has consistently been found.^2^

HLA-DR as a heterodimer molecule is composed of a highly polymorphic β-chain that is encoded by DRB1 or DRB3-5 genes. In most haplotypes, two HLA-DRB genes are expressed including one of the DRB1 locus and one of the loci encoding DRB3, 4 or 5. In the MS-associated HLA-DR15 haplotype, the two β-chains, HLA-DRB1*1501 and -DRB5*01 are resulted in two molecules that both are functional surface heterodimers and can serve as antigen presenting molecules for myelin basic protein (MBP)-specific T cells or autoreactive T cells that are highly cytotoxic.^3^

The HLA-DR15 gene is comprised of two DR β-chain molecules including HLA-DRB5*01:01 and HLA-DRB1*15:01. The region is in highly strong linkage disequilibrium (LD) (the inheritance of some genes together more than expected). The two β-chains as surface heterodimers include HLA-DRB1*1501 and DRB5*0101, which together with DR alpha result in functional surface molecules including DR2β (-DRA*0101, -DRB1*1501) and DR2α (-DRA*0101, -DRB5*0101). Whether both or only one of the two molecules contribute to etiology of MS is one of the most significant challenges in MS research. Based on studies, DR2α is an important etiologic risk factor of MS.^3^^-^^5^ Recent survey in humanized mice shows functional epistatic interactions whereby DRB5*0101 directly modulates the severity of MS through activation-induced cell death (AICD) of encephalitogenic T cells which are restricted by HLA-DRB1*1501 allele.^6^

According to a study, both DRB1*1501 and DRB5*0101 genes are always inherited together; it has not been possible until recently to determine conclusively which of them is the principal MS risk gene and what role each one plays. Furthermore, the processes that are induced by these molecules and resulted in MS development are unclear. However, they are signified in representation of myelin-derived molecules to CD4^+^ T cells. Indeed, it was shown that CD4^+^-MBP 85-99 complexes might be found in chronic active lesions of patients with MS.^7^

So far, several studies have been carried out on the association of DRB1*1501 and MS in Iran.^2^^,^^8^^-^^11^ Also, there is a high spectrum of variation in Khuzestan Province, Iran, in neighborhood of the Arabic countries and the Persian Gulf due to high migration rate from different ethnic populations.^12^^,^^13^ As there is no study so far about HLA-DRB1*1501-DRB5*01 correlation with the mentioned disease and given the ethnic variation as well as based on the fact that Iranian Arabs are mostly located in Khuzestan Province, this survey might be of great importance.

 We believe that our data on HLA haplotype will encourage future research to investigate the influence of this haplotype in MS pathogenesis. From this point of view, the present research aimed to use genotyping procedure to study association of HLA DRB1*1501-DRB5*01 and some of MS patients in Khuzestan Province. Furthermore, in the article, the probable relationship between this haplotype and relapsing-remitting MS (RRMS) course of disease, ethnicity, expanded disability status scale (EDSS),^14^ and gender was evaluated.

## Materials and Methods

Totally, 400 individuals participated in this study. Patients with MS of Khuzestan MS Society were diagnosed based on the McDonald criteria. Peripheral blood was collected from 200 patients. Neurological examination and routine laboratory data also were supplied. Informed consent was obtained from each participant and questionnaire was provided about features such as gender, race, familial history of MS, age, and MS course although clinical information was conducted by neurologist. The patients underwent thorough neurological laboratory tests. They were regularly examined and clinically followed up in the MS Society.

In the research, 200 healthy individuals without positive familial history of autoimmune disease were randomly selected. They referred to Shafa Hospital, Ahvaz City (the capital of Khuzestan Province) for routine laboratory tests and were registered as normal group. After obtaining informed consent, questionnaire form including information like sex, age, ethnicity as well as familial history of autoimmune disease was provided. Group matching for selection of controls was conducted to compare patients with controls on parameters like sex, age, and ethnicity. The origination and geographical zone of the both groups were the same.

Ethylenediaminetetraacetic acid (EDTA) tubes were used for collecting peripheral blood samples of control and patient cohort. Genome extraction was carried out by standard salting out protocol. Also electrophoresis and NanoDrop procedures were used for measuring the quantification and quantitation of extracted deoxyribonucleic acid (DNA); so, several randomly selected DNAs were used for the goal.

For genotyping of HLA, we employed polymerase chain reaction (PCR) amplification with sequence-specific primer (SSP) method (SSP-PCR method) which was repeated if unreliable findings were obtained.

The primers applied for amplification included the DRBI*1501 and DRB5*01 allele. Primer designing was carried out using the IMGT/HLA database (http://www.ebi.ac.uk) and alignment was obtained in NCBI/blast (www.ncbi.nlm.nih.gov).

Forasmuch as every variant is assigned by the presence of specific band on gel electrophoresis, the size of product might be useful in the data interpreting.

Every SSP-PCR is assumed to work successfully if the positive control is amplified. Myelin oligodendrocyte glycoprotein (MOG) gene was positive control for each reaction. So in appropriate circumstance, a band of 356 bp must be detected. The MOG and DRBI*1501 primers were demonstrated in a previous study.^2^ The result validation was done by sequencing selected samples randomly.

Agarose gel electrophoresis was applied for detection of PCR products. Laboratory tests were conducted in the Genetic Laboratory of Shahid Chamran University of Ahvaz.

The HLA-DRB1*1501 and HLA-DRB5*05 frequency were already published.^2^^,^^17^ The percentage of HLA-DRB1*1501-DRB5*05 haplotype was calculated. Fisher’s exact and chi-square tests were used for comparison of the named haplotype percentage in the case and control groups. Statistical analyzing of data was applied using SPSS software (version 16, SPSS Inc., Chicago, IL, USA). An odds ratio (OR) with 95% confidence interval (CI) and a P-value of < 0.050 were considered significant.

## Results

In total, 400 individuals were investigated in this study including 200 patients with MS and 200 healthy blood donors from Khuzestan Province. Frequency of the haplotypes regarding HLA-DRB1*1501 and -DRB5*01 was measured precisely.

Features of the patients with MS were published elsewhere.^2^^,^^15^

Frequency and association of DRB1*1501^+^-DRB5*01^+^, DRB1*1501^+^-DRB5*01^-^, DRB1*1501^-^-DRB5*01^+^, and DRB1*1501^-^-DRB5*01^-^ haplotypes were calculated in the patients and compared with controls (Table 1). Findings revealed that the percentage of DRB1*1501^+^-DRB5*01^-^ **(**29.73% vs. 11.81%, P < 0.001, OR = 3.159, 95% CI = 1.692-5.898) increased signiﬁcantly among the patients rather than controls, statistically. Results showed that frequency of DRB1*1501^-^-DRB5*01^-^ reduced meaningfully in patients, hence negative association was observed **(**42.16% vs. 68.50%, P < 0.001, OR = 0.335, 95% CI = 0.208-0.539). In other words, absence of both loci is associated with healthy group, although no meaningful association was revealed between DRB1*1501^+^-DRB5*01^+^ (12.97% vs. 7.87%, P = 0.156, OR = 1.744, 95% CI = 0.803-3.783) and DRB1*1501^-^-DRB5*01^+^ (15.13% vs. 11.81%, P = 0.403, OR = 1.332, 95% CI = 0.680-2.608) haplotypes and MS risk (Table 1).

**Table 1:** Distribution analysis of human leukocyte antigen (HLA)-DRB1*1501-DRB5*01 haplotypes in patients with multiple sclerosis (MS) and controls

**Haplotypes**	**Positive haplotype**		**OR**	**95% CI**	**P**
	**Patients (n = 200)**	**Controls (n = 200)**			
	**n (%)**	**n (%)**			
DRB1*1501^+^-DRB5*01^+^	24 (12.97)	10 (7.87)	1.744	0.803-3.783	0.156
DRB1*1501^+^-DRB5*01^-^	55 (29.73)	15 (11.81)	3.159	1.692-5.898	< 0.001*
DRB1*1501^-^-DRB5*01^+^	28 (15.13)	15 (11.81)	1.332	0.680-2.608	0.403
DRB1*1501^-^-DRB5*01^-^	78 (42.16)	87 (68.50)	0.335	0.208-0.539	< 0.001*

OR: Odds ratio; CI: Confidence interval

* Significant P-values; statistical significance was at P < 0.050.

**Table 2:** Association of human leukocyte antigen (HLA) haplotypes and female patients with multiple sclerosis (MS) in Khuzestan, Iran

**HLA haplotypes**	**Positive haplotype**		**Total**		**P**
	**Patients [n (%)]**	**Controls [n (%)]**	**Patients**	**Controls**	
DRB1*1501^+^-DRB5*01^+^	19 (12.02)	11 (18.03)	158	61	0.246
DRB1*1501^-^-DRB5*01^-^	62 (41.33)	34 (52.30)	150	65	0.137
DRB1*1501^+^-DRB5*01^-^	43 (27.21)	13 (20.00)	158	65	0.259
DRB1*1501^-^-DRB5*01^+^	23 (16.19)	6 (6.12)	142	98	0.019*

HLA: Human leukocyte antigen

* Significant P-values; statistical significance was at P < 0.050.

Furthermore, association of the mentioned haplotypes with MS for both genders was analyzed, separately. As it was shown in tables 2 and 3, no association between the haplotypes and any gender, except for DRB1*1501^-^-DRB5*01^+^, was found. It showed meaningful difference with women (16.19% vs. 6.12%, P = 0.019). Furthermore, no positive association was found between the mentioned haplotypes and both ethnicities (Arab and Persian) (Tables 4 and 5), though significant association was shown between DRB1*1501^-^-DRB5*01^+^ haplotype and Persian patients (17.11% vs. 5.79%, P = 0.027) (Table 5).

There are four types of MS including RRMS, secondary-progressive MS (SPMS), relapsing-progressive MS (RPMS), and primary-progressive MS (PPMS). Clinical course of MS and EDSS score were diagnosed by neurologist. As more than 80% of the patients with MS were of RRMS type, so they were divided into two groups: RRMS and non-RRMS. EDSS scale was measured in 0-10 range. Most of the patients had less than 5 EDSS steps; so the patients were stratified into two groups based on the EDSS steps including 1-4.5 and 5-10. Then, haplotype associations with MS course and EDSS were estimated. DRB1*1501^+^-DRB5*01^-^ haplotype was strongly associated with EDSS score of 5-10 (62.50% vs. 25.76%, P = 0.026) though rest of the haplotypes were not positively associated with the level of disability (data is shown in table 6). None of the mentioned variants demonstrated significant correlation with RRMS as the most common disease course (Table 7).

## Discussion

Epidemiology and genetic sciences have consistently identiﬁed association of HLA-II genes with most of the autoimmune diseases. MS as an autoimmune disease is targeting myelin of the CNS. The DR2 haplotype (DRB1*1501^+^-DRB5*0101^+^-DQB1*0602^+^) is identiﬁed as strongest studied genetic risk factor in Caucasians.

A study on Khuzestan population showed no association between MS risk and DRB5*0101.^15^ In a survey in Sweden, it was shown that the HLA haplotype involved in MS is the DRB5*0101 allele.^16^ A positively associated HLA-DRB5 locus with African-American patients with MS was reported.^17^ Moreover, a significant association with DRB5 variants among Brazilian patients with MS was measured.^18^ Contrary to this, no association between DRB5*0101 and Japanese patients with MS was shown.^19^ However, in the current study, DRB1*1501^-^-DRB5*01^+^ showed no association with MS. These observed differences might be because of a high spectrum of various ethnic backgrounds in Khuzestan which is in neighborhood of the Persian Gulf and Arabic countries and might be due to high migration rate from different ethnicities.

**Table 3:** Association of human leukocyte antigen (HLA) haplotypes and male patients with multiple sclerosis (MS) in Khuzestan, Iran

**HLA haplotypes**	**Positive haplotype**		**Total**		**P**
	**Patients [n (%)]**	**Controls [n (%)]**	**Patients**	**Controls**	
DRB1*1501^+^-DRB5*01^+^	5 (13.51)	1 (6.25)	37	16	0.444
DRB1*1501^-^-DRB5*01^-^	19 (50.00)	8 (57.14)	38	14	0.647
DRB1*1501^+^-DRB5*01^-^	11 (29.72)	6 (35.29)	37	17	0.683
DRB1*1501^-^-DRB5*01^+^	4 (9.75)	1 (3.12)	41	32	0.266

HLA: Human leukocyte antigen

Statistical significance was at P < 0.050.

**Table 4:** Analysis of association between human leukocyte antigen (HLA) haplotypes and Arab ethnicity of Khuzestan, Iran

**HLA haplotypes**	**Positive haplotype**		**Total**			**P**
	**Patients [n (%)]**	**Controls [n (%)]**		**Patients**	**Controls**	
DRB1*1501^+^-DRB5*01^+^	7 (9.85)	9 (16.98)		71	53	0.243
DRB1*1501^-^-DRB5*01^-^	34 (48.57)	25 (43.10)		70	58	0.537
DRB1*1501^+^-DRB5*01^-^	19 (27.14)	11 (26.19)		70	42	0.912
DRB1*1501^-^-DRB5*01^+^	7 (9.58)	3 (4.05)		73	74	0.183

HLA: Human leukocyte antigen

Statistical significance was at P < 0.050.

So far, studies about DRB1*1501 and DRB5*01 allele have been done on Khuzestan 

population.^2^^,^^15^^,^^20^ In a study, it was shown that significant difference in the frequencies of DRB1*1501 was detected between patients with MS and control population of Khuzestan.^2^ However, HLA-DRB5*01 and MS risk of this population had not any association.^15^ Also, we found association between DRB1*1501^+^-DRB5*01^-^ and susceptibility to MS in this study. The results are in line with this fact that HLA DRB1*1501 has important role in associating with MS and it is confirmed by studies conducted before.^20^^,^^21^

Another recent study on normal population of Khuzestan reported that DRB1*1501^+^-DQB1*0602^+^-DQA1*0102^+^-DRB5*01^-^, DRB1*1501^-^-DQB1*0602^+^-DQA1*0102^+^-DRB5*01^-^, DRB1*1501^+^-DQB1*0602^+^-DQA1*0102^+^-DRB5*01^+^, and DRB1*1501^-^-DQB1*0602^+^-DQA1*0102^+^-DRB5*01^+^ haplotypes were the most frequent four allelic haplotype in this population.^22^ We found no association between DRB5*01^+^-DRB1*1501^+^ haplotype and MS in this study. Based on the recodes, this putative haplotype has normally high frequency in this population. While DRB1*1501^+^-DQB1*0602^+^-DQA1*0102^+^-DRB5*01^-^ and DRB1*1501^-^-DQB1*0602^+^-DQA1*0102^+^-DRB5*01^-^ have high frequencies in the normal population,^22^ but it was observed that DRB1*1501^+^-DRB5*01^-^ and DRB1*1501^-^-DRB5*01^-^ distribution were not the same between patient and control groups. It shows that they might be frequent haplotypes in Khuzestan population although carrying them increases the risk of MS developing. 

In a study on MS, DR2 haplotypes have generally received most attention as they are present in 60% of Caucasian patients with MS but only in 20%–30% of unaffected individuals. DR2 haplotype contains three MS-associated genes: DRB5*0101, DRB1*1501, and DQB1*0602, that encode β-chains for the HLA class II molecules including DR2a, DR2b, and DQ6, respectively. In all ethnic groups studied so far, DRB1*1501 and DRB5*0101 are approximately inseparable. The LD is less pronounced for DQ6; in African populations, DQ6 is sometimes detected without DR2α or DR2β and does not then make prone to MS. Some association studies indicate that DRB5*0101 and DRB1*1501 are the primary MS risk genes independent of DQB1*0602.^23^^,^^24^

In most populations, DRB1*1501, DRB5*0101, and DQB1*0602 genes are in strong LD. However, in African Americans, the DRB1*1501 and DRB5*0101 alleles do not display the high degree of LD with the DQB1*0602 variant that is the characteristic of Europeans. A study on a large African-American group revealed that having DRB1*15 not DQB1*0602 allele strongly increased in patients with MS, whereas carrying the DQB1*0602 allele with other DRB1 alleles (i.e., not DRB1*15 alleles) was present at identical frequencies in patients and controls.^23^ An association research revealed that DRB1*1501, rather than DQB1*0602, constituted the principal MS disease risk gene.^23^

In studies on Northern European Caucasian populations, association of DRB1*1501^+^-DRB5*0101^+^-DQB1*0201^+^ haplotype with MS was observed.^25^^,^^26^

**Table 5:** Analysis of association between human leukocyte antigen (HLA) haplotypes and Persian ethnicity of Khuzestan, Iran

**HLA haplotypes**	**Positive haplotype**		**Total**		**P**
	**Patients [n (%)]**	**Controls [n (%)]**	**Patients**	**Controls**	
DRB1*1501^+^-DRB5*01^+^	14 (12.17)	6 (14.63)	115	41	0.686
DRB1*1501^+^-DRB5*01^-^	34 (29.56)	7 (19.44)	115	36	0.233
DRB1*1501^-^-DRB5*01^+^	19 (17.11)	4 (5.79)	111	69	0.027*
DRB1*1501^-^-DRB5*01^-^	44 (38.59)	20 (41.66)	114	48	0.715

HLA: Human leukocyte antigen

* Significant P-values; statistical significance was at P < 0.050.

**Table 6:** Association of human leukocyte antigen (HLA) haplotypes with expanded disability status scale (EDSS) in patients with multiple sclerosis (MS) in Khuzestan, Iran

**HLA haplotypes**	**Positive haplotype**		**Total**		**P**
	**EDSS (1-4.5)**	**EDSS (5-10)**	**EDSS (1-4.5)**	**EDSS (5-10)**	
	**n (%)**	**n (%)**	**n (%)**	**n (%)**	
DRB1*1501^+^-DRB5*01^+^	22 (13.58)	1 (12.50)	162	8	0.931
DRB1*1501^-^-DRB5*01^-^	67 (40.85)	2 (33.33)	164	6	0.713
DRB1*1501^+^-DRB5*01^-^	42 (25.76)	5 (62.50)	163	8	0.026*
DRB1*1501^-^-DRB5*01^+^	25 (15.92)	0 (0)	157	8	0.220

HLA: Human leukocyte antigen; EDSS: Expanded disability status scale

* Significant P-values; statistical significance was at P < 0.050.

Findings of a study showed that both DRB5*0101 and DRB1*1501 expression happened 

in the HLA-DR15 haplotype that was associated with MS risk. Also, it was found that transcripts of DRB5*0101 allele were more common in all tissues; accordingly, DRB5*0101 and DRB1*1501 expression were at substantial and largely comparable levels on the surface of various cells; also their expression was similarly modulated upon activation of B cells and monocytes by interleukin-4 (IL-4).^3^ Furthermore, in a study on Brazilian patients with MS, overrepresentation of DRB5 was reported.^18^^,^^26^

Percentage of carrying DRB1*1501 and DRB5*0101 alleles, which have been proved to be associated with MS in many studies, was higher in Japanese patients with MS than in control groups, though the comparable level was not significant. Moreover, DRB5*0101 allele showed positive association with opticospinal MS (OSMS).^18^^,^^19^

In another study, it was revealed that the MS-associated haplotype and narcolepsy could thus be specified as DRB1*1501, DRB5*0101, DQA1*0102, DQB1*0602; also the association to DRB5*0101 was as strong as the association to the DRB1*1501 allele.^16^

Finally, goal of the present survey was to demonstrate a part of MS genetic background in Khuzestan Province. MS is an autoimmune disease caused by interaction of genetic and environmental features. It shares features that suggest common pathogenesis pathways. There are several genes involved in the pathogenesis of the multifactorial disease. Thus, it is possible that other haplotypes, variants, and mutations in other genes are associated with MS susceptibility. Therewith, differences observed between results in different populations might be caused by discrepancy in the gene pool, sample size, environmental factors, and lifestyles. Lack of large sample size might have influence on the power of statistical analysis. Conclusively, our results need to be verified in bigger population assays. Moreover, the heterogeneous population includes different ethnicities like Lur, Arab, Kurd, Turk, and Fars. Therefore, the research gives further support to the significance of replication studies as susceptible loci that may be various in different ethnicities. For obtaining reliable data, it is recommended to study genotype different variants in Iranian MS population of other provinces and MS population of other countries, chiefly in high-risk regions for MS. Unfortunately, we found no more data regarding the association of the haplotypes that were investigated in this study with MS, MS course, EDSS, gender, and ethnicity. So, we strongly suggest investigating the association of mentioned haplotypes with MS, clinical course, EDSS, sex, and race.

**Table 7:** Analysis of association between human leukocyte antigen (HLA) haplotypes with relapsing-remitting multiple sclerosis (RRMS)

**HLA haplotypes**	**Positive haplotype**		**Total**		**P**
	**RRMS [n (%)]**	**Non-RRMS [n (%)]**	**RRMS**	**Non-RRMS**	
DRB1*1501^+^-DRB5*01^+^	22 (13.75)	1 (11.11)	160	9	0.822
DRB1*1501^-^-DRB5*01^-^	69 (42.33)	4 (44.44)	163	9	0.901
DRB1*1501^+^-DRB5*01^-^	48 (27.58)	3 (33.33)	174	9	0.708
DRB1*1501^-^-DRB5*01^+^	24 (14.11)	1 (11.11)	170	9	0.800

HLA: Human leukocyte antigen; RRMS: Relapsing-remitting multiple sclerosis

Statistical significance was at P < 0.050.

## Conclusion

Our results indicate that DRB1*1501^+^-DRB5*01^-^ and DRB1*1501^-^-DRB5*01^-^ haplotypes may have correlation with MS risk. In addition, our findings suggest that HLA-DRB1*1501^-^-DRB5*01^+^ is involved in MS susceptibility among women and Persians. DRB1*1501^+^-DRB5*01^-^ genotype frequency may play a key role in MS developing.

## Notes:

**How to cite this article:** Delfan N, Galehdari H, Shafiei M, Ghanbari-Mardasi F, Latifi T, Majdinasab N, et al. Association of human leukocyte antigen-DRB haplotype in multiple sclerosis population of Khuzestan, Iran. Iran J Neurol 2018; 17(4): 154-60.
